# Utilities of the Post-anesthesia State derived by the Standard Gamble method in surgical patients

**DOI:** 10.1186/1472-6947-6-8

**Published:** 2006-02-15

**Authors:** Saifudin Rashiq, Diane Edlund, Bruce D Dick

**Affiliations:** 1 Department of Anesthesiology & Pain Medicine, University of Alberta, 8-120 Clinical Sciences Building, Edmonton, AB T6G 2G3, Canada

## Abstract

**Background:**

There are no published utilities for the post-anesthesia state obtained by the standard gamble method (SG).

**Methods:**

We obtained utilities for postoperative pain, nausea, vomiting, urinary retention and myalgia from 100 adults prior to elective surgery using SG.

**Results:**

20% of volunteer participants could not demonstrate a satisfactory understanding of the SG process. Median utilities for each adverse effect were all very close to 1.0, and no statistically significant differences were found between them.

**Conclusion:**

Our results suggest that the avoidance of anesthesia related side effects and pain is not viewed by patients prior to surgery as being worthy of the taking of even a miniscule risk of death. This may affect the decision to utilize anesthesia techniques that trade a lower incidence of common side effects for a very low but finite risk of a catastrophic complication.

## Background

Anesthesia can be given in a wide variety of different ways. Many operations can either be performed under general anesthesia, regional anesthesia (targeted blockade with local anesthetic of the nerves supplying the area to be operated on) or local anesthesia. Even within these broad classifications, a number of drugs, techniques and routes of administration may be used alone or together in a large number of potential combinations. Anesthesiologists tailor each anesthetic to the specific requirements of the case by considering factors such as the magnitude and duration of the procedure, the severity of co-morbidities exhibited by the patient, the anticipated degree of postoperative pain and nausea and the desirability of same-day home discharge, among other things. Advances in drugs, equipment and training have combined to make anesthesia a very safe process, and most anesthesia research is now focused on reducing the impact of transient adverse effects, rather than on reducing the incidence of serious injury and death (Chung & Mezei 1999). Nonetheless, the rates of catastrophic complication related to anesthesia have not been reduced to zero (Kaufman et al 2000).

Each anesthetic drug or technique choice carries its own benefits and risks and there is currently no quantitative method for distinguishing between them. The selection of anesthetic technique would therefore seem to an appropriate area for the application of decision analysis. Decision support tools might, for example, help decide if the reduced risk of perioperative pain and vomiting afforded by regional anesthesia over general anesthesia is justified in the face of the small attendant risk of permanent neurologic injury. The construction of such decision aids would require estimates of both the probability and the utility of each adverse outcome under consideration.

While relatively good estimates are available for the probability of common and rare adverse anesthesia effects, there are no formally derived utility estimates. Instead, researchers have used other methods to enable subjects to rank and quantify the degree to which they would choose to avoid them. For example, our group recently performed a study of the 'willingness to pay' type, (Rashiq & Bray 2003), in which pre-surgical patients were asked to allocate 100 theoretical dollars to each of ten post-operative states in proportion to the importance they attached to them. Pain was ranked most unpleasant by 49% of participants, and they allocated a median of $25 to avoid it. Avoiding vomiting was ranked most unpleasant by 24% of participants, with a median of $20 allocated to avoiding it.

The difficulty with this method of preference derivation is that it does not incorporate consideration of risk. Utilities, which are derived in the context of risk, are felt to better reflect subjects' true feelings since, in real life, treatment decisions are only made after attendant risks have been considered. Utilities may be derived by more than one method. However, the only available utility values for anesthesia-related effects were not derived empirically but assigned by a panel of experts. Using this method, post-operative nausea and vomiting was assigned a utility of 0.72, a figure that we thought to be implausibly low. (Reeves et al 2001). The Standard Gamble (SG) is felt by many to be the gold standard method of utility derivation, but SG has not hitherto been applied to anesthesia side effects.

The primary objective of this study was to obtain utilities for five common short-term complications of anesthesia and surgery using the standard gamble (SG) method in 100 patients presenting for elective surgery at our institution. A second objective was to compare the age, gender and educational level of subjects who could not successfully complete the SG interview with those who did so. Finally, we wished to compare the rank order of these effects for the group as a whole with that obtained by our previous willingness to pay study.

## Methods

The University of Alberta Hospital is a tertiary referral centre in Western Canada with a surgical practice profile that is skewed towards complex procedures and patients with a high degree of concomitant medical illness. Almost all patients who present for elective surgery are evaluated in a Preadmission Clinic at least one day prior to admission.

Having obtained the approval of the Health Research Ethics Board of the University of Alberta, a single, trained interviewer invited a random sample of each day's Preadmission Clinic attendees to participate in the study. The only inclusion criteria were that candidate subjects be adults (18–65 years) who were being pre-admitted for elective surgery. Subjects who were cognitively or visually impaired, not fluent in written and spoken English, or too anxious about forthcoming events to participate were excluded.

Informed written consent was obtained from each participant. Interviews were conducted in a quiet room. Subject age, gender, proposed surgical procedure and highest completed educational level were recorded. A standardized explanation of the SG lottery was given using a chart designed by the investigators. This chart consisted of a line diagram of an individual walking down a path which forks two ways, with an illustration of the consequences of going down each path (figure).

The process began with the description of two hypothetical disease states. The first was intended to represent an incurable, severely physically and socially debilitating condition and the second a trivial illness with little symptom burden that could be easily controlled (Table 1, first two rows). Subjects completed SG lotteries for each of these using certain death probabilities ranging from 1 in 1,000,000,000 to 1 in 10. We began with the lowest risk of death and worked sequentially upwards, unless the subject elected to jump ahead without prompting. Every effort was made to ensure that subjects understood the process but the interviewer was careful not to coach the subject towards or away from any particular decision. The diagram was used for each lottery and while the subject was considering his or her decision, the death probability in play was kept in view. Subjects who could not complete the lottery, those who gave the same utility to both health states, and those who rated the severe illness as having a higher utility than the trivial one were deemed to have an incomplete understanding of the process. Their data were excluded from further analysis. We also excluded anyone who completed this stage but who did not give an indifference probability in at least one of the five lotteries in the subsequent phase of the study.

**Table 1:** Descriptions of the hypothetical illnesses and postoperative adverse effects used

State/effect	Description
Trivial hypothetical illness	You have an itchy red skin rash on your back. Your clothes cover it up and if you put special cream on it once a day, you feel fine. There is no scarring. There is no cure, but the cream is safe to use forever.
Debilitating hypothetical illness	You always feel very cold, no matter what the weather. You spend the whole day in bed, wearing all your clothes, constantly shivering. Your teeth chatter constantly so that it's impossible for you to speak clearly. You can't work, use the telephone or drive a car. People stare at you in public, so you don't go out. There is no cure or treatment for this disease.
Myalgia	It is the day after your surgery. All your muscles ache for the whole day, as if you had flu. The ache gets worse when you try to move, but you manage to get around
Nausea	You are lying on your side, awake and aware of your surroundings in the recovery room. You are extremely queasy, as if you were on a boat in rough seas. The least movement makes the nausea worse
Pain	You are lying on your back, awake and aware of your surroundings in the recovery room. Your surgical incision really hurts, as if a knife was stabbing you. Movement makes the pain worse, and no position seems to make it better
Urinary retention	You are in the recovery room, awake and alert. You want to pass urine (water) but no matter how hard you try, none comes out
Vomiting	You are lying on your side, awake and aware of your surroundings in the recovery room. You feel waves of nausea and are throwing up. Your abdominal and chest muscles ache from vomiting

Subjects who successfully completed the lottery for the two theoretical illnesses participated in the rest of the study. Recruitment continued until 100 such subjects had been collected. In this phase, short descriptions of five common adverse effects that might be experienced following anesthesia and surgery were read to the patient and also given to them in writing. These descriptions were derived for our previous work by a focus group of anesthesia providers (Table 1) Subjects then participated in individual SG lotteries for each effect. Death probability iterations were varied in the same way as before but it was made clear to subjects that they could choose any numerical probability they wished to decide their true point of indifference. The probability of death at the point of indifference was subtracted from 1.0 to yield the utility of the state. For example, if a subject was indifferent to the choice between the adverse anesthesia effect as described and a lottery with a 1% risk of death and a 99% chance of avoiding the effect, the derived utility would be 0.99. If even the smallest risk of death was not acceptable to the subject for a given adverse effect, we assigned a utility of 1.0 for the state.

Data was recorded by hand for subsequent transfer to a Microsoft Excel spreadsheet. All data sheets were checked twice for coding errors. Statistical analysis was performed using SAS version 8 for Windows. An analysis of variance (performed on a reciprocal transformation) was used to seek differences between the utilities for the various states. Chi-square tests were used to compare the gender and educational level of those who completed the study vs. those who were excluded, and a t-test was used to compare the ages of these two groups. Statistical significance for test probabilities was pre-determined to be 0.05.

## Results

121 surgical patients agreed to participate in the study. Of these, 21 were excluded, either because they did not give an appropriate response to the screening lotteries (16) or were unable to complete the protocol for some other reason (5). Those excluded were not different in age, gender or educational level from those that went on to complete the study. Thus, 100 subjects completed the study by providing indifference probabilities for some or all the adverse effects under investigation. Demographic and other characteristics of this group are given in Table 2.

**Table 2:** Demographic characteristics of the subject group

**Variable **(n = 100)	n, %
Age (yr: mean ± SD)	49 ± 12
Male %	62
Highest Educational level attained:	
Less than Grade 12	21
Grade 12	28
At least some college or trade school	14
At least some university	36
Not recorded	1
Surgical subspecialty:	
General	32
Cardiac/vascular	18
Neurosurgery	16
Urology	8
Thoracic	7
Orthopedic	7
ENT	7
Plastic	5

Five adverse effect-specific lotteries were attempted for each subject for a total of 500 lotteries. In 117 of these (23%), the subject was unwilling to take any risk of death, even when explicitly asked to choose a value smaller than 1: 100 000 000. We assigned a utility of 1.0 in these situations. There was no difference in age, gender or educational level between those who provided a non-zero risk in all 5 lotteries versus those who did so in four or fewer.

The distribution of utilities is given in Table 3. There was no statistically significant difference between them. As expected, the highest utility was given to the trivial hypothetical illness, and the debilitating hypothetical illness was given the least utility. The adverse effect with the lowest utility was pain in 35 subjects, vomiting in 30, urinary retention in 28, nausea in 4 and myalgia in 3. There was no difference between the genders for any of the individual utility estimates.

**Table 3:** Derived utilities for each state

	Minimum	Mean	Median	Maximum
Trivial hypothetical illness	0.900000	0.997974	0.999965	1.000000
Myalgia	0.500000	0.988778	0.999990	1.000000
Nausea	0.500000	0.977719	0.999900	1.000000
Vomiting	0.500000	0.957954	0.999900	1.000000
Urinary retention	0.500000	0.957253	0.999800	1.000000
Pain	0.500000	0.943390	0.999817	1.000000
Debilitating hypothetical illness	0.500000	0.773357	0.960000	1.000000

## Discussion

We have elicited utilities for five adverse effect states that are commonly found after anesthesia and surgery. This is the first time, to our knowledge, that the disutility of perioperative morbidity has been measured directly using the SG method.

The SG method has been widely used to measure individual preferences in conditions of uncertainty (Gafni, 1994). The utilities thus derived take potential health states and quality-adjusted years of life following medical procedures into account (Farquhar, 1984. This method has been widely used in health care practice for decades (Torrance, 1986), providing a valuable index that is useful in health care decision making processes.

Our first finding was that almost 20% of those who agreed to participate in our study could not satisfactorily complete the screening test. We took this to mean that they did not fully understand the SG exercise. An alternative interpretation is that they really did think that the two hypothetical disease states would be equally burdensome, or our 'trivial' hypothetical illness was worse than the 'debilitating'. We think this interpretation implausible, but if it were true, we would have unwittingly selected out respondents who characterize illness burden in this highly unconventional way. We did not find any differences in age, gender or educational level between those who went on to complete the study and those who did not and must therefore conclude that another factor or factors that we did not measure is responsible for this difference. Approximately half of our sample had at least some post-secondary education, which is broadly in line with the Canadian population as a whole.

The abstract and cognitively demanding nature of the SG has been noted by others (Gold et al 1996), and has been cited as a threat to the validity of its results. An alternative viewpoint is that if it is carefully explained and illustrated visually, as we did, the SG is a feasible method of obtaining utilities in both sick and healthy people, and that the additional inconveniences it imposes on interviewer and respondent are outweighed by its superior theoretical basis (Torrance 1986). We selected the SG for its theoretical benefits alone.

For almost all subjects, the disutility of the adverse effects was very low. They placed little value in absolute terms in avoiding them. The median utility for postoperative vomiting, for instance, was 0.999900. This calls into question the assumption that was made in a previous decision analysis of anesthesia for cataract surgery, in which the utility of postoperative nausea and vomiting was estimated by an expert panel of health care providers to be 0.72 (Reeves et al 2001). Our data are more consistent with the results of a large-scale community based survey of the general public in Canada (Mittmann et al 1999), which gave average utilities for common chronic health conditions on a 0–1 scale. In that study, disease-specific utilities ranged from 0.92 for acne to 0.58 for Alzheimer's disease. In that context, the allocation by our subjects of much greater utilities to temporary anesthesia and surgery-related side-effects, even if severe and debilitating makes sense.

A factor that may have contributed to these results is the SG itself: individuals have been found to attach a higher preference to avoiding a loss than to making a gain, even if the loss and gain are of the same magnitude (Tversky and Kahneman, 1981). While more recent theories of decision-making processes offer more complex models to explain these processes (e.g., Busemeyer et al., 2005), loss aversion remains an evidence-based theory (Usher and McClelland, 2004). Utilities derived by SG therefore likely reflect this innate risk-aversion, resulting in higher scores than other methods. This is exemplified by a study in which the simultaneous use of SG and the Rating Scale to derive utilities in chronic musculoskeletal pain yielded reproducibly higher utilities by SG (Goosens et al 1999). We may have obtained higher utilities by our interactive method than would have been obtained by an alternative method such as a self-completed questionnaire (Hammerschmidt et al 2004). We offered risks in sequential order from lowest to highest, rather than a 'ping-pong' approach (offering a very low risk, then a very high one and going back and forth to find the point of indifference). We did this because we thought it would be less overwhelming for our subjects. However, if the subject ended the lottery prematurely because (s)he was tired or uncertain, our method would have biased the result towards higher utilities.

We were unable to demonstrate statistically significant differences between the utilities for any of the states we described. This may in part reflect a ceiling effect. While the rank order of the utility for the adverse effects is not completely consistent with our previous work in the area (Rashiq and Bray 2003), pain was the state to which participants attached the lowest utility in both studies, and they did so in the same proportions to that which was found previously by others (Gan et al 2001, Macario et al 1999) using different methods of preference derivation.

Our use of death as the anchor for the low end of the utility scale may be questioned. The use of an alternative reference state would likely have yielded lower utilities (Torrance 1986). However, recognized adverse effects of commonly performed anesthesia procedures include not only transient phenomena such as nausea and pain but very rare catastrophes. Peripheral nerve block, performed thousands of times daily in operating rooms worldwide is documented to have caused nerve damage leading to lifelong and severe disability (Kaufman 1999). The incidence of permanent paraplegia following regional anesthesia is estimated to be of the order of 1 in 10 000 (Sage 1999). Most anesthesiologists performing regional anesthesia will therefore never be associated with such a case in their entire careers and many feel that the benefits of regional anesthesia (less pain, nausea, and a more rapid convalescence, for example) outweigh its risk in many circumstances. However, if a decision analysis were to be used to decide whether regional or general anesthesia should be used to avoid postoperative nausea, using a published utility for paraplegia of 0.329 (Ustun 1999) and our utility for postoperative nausea of 0.999900, we think that the rationale for this choice would be less clear.

## Conclusion

We conclude that it is possible to obtain utilities for the post-anesthesia state by the SG method albeit that a significantly large subgroup (23%) of people asked will not be able to give meaningful responses. We did not demonstrate any differences in age, gender or education between those who completed the study and those who did not. The rank order of utilities for the adverse effects was somewhat different from that which we obtained by the willingness-to-pay methodology in a previous study, but pain and vomiting were still the effects rated as least desirable.

We find the utility of common post-anesthesia side effects to be very high, and suggest that this finding would not support the use of anesthesia techniques that carry even a very small risk of catastrophic complication in decision analysis models, even if those techniques effectively reduce common side-effects.

## Competing interests

The author(s) declare that they have no competing interests.

## Authors' contributions

SR conceived the study idea. All authors designed the study. DE performed the data collection. SR and BDD performed the data analysis. SR and BDD wrote the manuscript and DE read, amended and approved the final draft.

## Pre-publication history

The pre-publication history for this paper can be accessed here:


